# Gene therapy in hemophilia: the dawn of a new era

**DOI:** 10.1016/j.rpth.2024.102640

**Published:** 2024-11-28

**Authors:** Roberta Gualtierotti, Andrea Giachi, Niccolò Bitto, Vincenzo La Mura, Flora Peyvandi

**Affiliations:** 1 Dipartimento di Fisiopatologia Medico-chirurgica e dei Trapianti, Università degli Studi di Milano, Milano, Italia; 2 Centro Emofilia e Trombosi Angelo Bianchi Bonomi e S.C. Medicina - Emostasi e Trombosi, Fondazione Istituto di Ricovero e Cura a Carattere Scientifico Ca' Granda Ospedale Maggiore Policlinico, Milano, Italia

**Keywords:** disease management, gene therapy, hemophilia, liver, multidisciplinary care team

## Abstract

Hemophilia A and B are hereditary bleeding disorders associated with the X chromosome, stemming from genetic defects in the coding of coagulation factor (F)VIII or FIX protein, leading to partial or complete deficiency. In the absence of effective prophylaxis, these deficiencies can result in irreversible joint damage, known as hemophilic arthropathy, and subsequent disability.

Despite advancements in hemophilia treatment, individuals with severe forms of the disease continue to face a high risk of bleeding, particularly in instances of trauma or major surgical procedures. In such scenarios, it remains imperative to administer replacement or bypassing drugs, especially when inhibitors are present.

Within this context, gene therapy emerges as a compelling alternative, ensuring sustained expression of the deficient factor at levels often surpassing current recommendations. Some studies report an effect lasting up to 8 years, contributing significantly to clinical improvement and enhancing the quality of life for patients. However, a comprehensive evaluation of this innovative therapy is essential, encompassing both its benefits and potential risks. It is crucial to undertake a multidisciplinary assessment, engage in thoughtful discussions with the patient, and closely monitor the therapy’s effects and any eventual side effects of therapy. This approach aims to facilitate an informed and collaborative decision-making process, ultimately maximizing the benefits for each individual patient.

## Essentials

• Hemophilia A and B are genetic disorders causing bleeding tendency due to missing proteins.
• Without preventive care, hemophilia causes joint damage and disability due to repeated bleeding.
• Gene therapy offers long-lasting improvements, although it may also lead to severe side effects.
• Multidisciplinary and patient discussions are vital to balance gene therapy benefits and risks.

## 1 Introduction

Hemophilia A and B are congenital and hereditary bleeding disorders linked to the X chromosome, caused by defects in genes coding for coagulation factor (F)VIII and FIX proteins, resulting in partial or total deficiency [1]. Children and adults, especially those with severe hemophilia (defined as FVIII or FIX levels <1 U/dL), if not appropriately treated with adequate prophylaxis with replacement or nonreplacement drugs, may experience spontaneous life-threatening bleeding such as intracranial hemorrhages or musculoskeletal bleeding episodes, which account for 80% of cases, mainly in the form of intra-articular bleeding (hemarthrosis), occurring more frequently in ankles, elbows, and knees or severe muscle hematomas, such as in the iliopsoas muscle [2]. In the absence of adequate prophylaxis, this can lead to irreversible joint damage (hemophilic arthropathy) and disability [3].

While a minimum trough plasma factor level of 1% was recommended some years ago, Manco-Johnson et al. [4] demonstrated that patients treated with prophylaxis with a FVIII trough level > 1% may still develop subclinical joint damage. For this reason, current recommendations from European and global scientific societies advise a minimum target of 3% to 5% trough level of the deficient factor [5,6]. However, maintaining these trough levels with a standard half-life factor requires every other day administration for hemophilia A and around twice a week administration for hemophilia B. Therefore, in the last decade, efforts were directed toward reducing the number of infusions, obtaining higher trough levels, fewer annual bleeding episodes, and subsequently increased protection for patients, which was achieved with the introduction of extended half-life replacement FVIII or FIX drugs [7,8]. However, the number of intravenous infusions required by the current replacement drug prophylaxis regimen remains still high, especially for most extended half-life FVIII, administered at most every 4 to 5 days, and in the case of more recent FVIII products every week, negatively impacting the patient’s quality of life and treatment adherence [5,9,10]. Similarly, nonreplacement therapies, such as the commercially available emicizumab, have transformed the management of persons with severe hemophilia A, initially for those with inhibitors and later for those without, offering particular benefits for patients with limited venous access due to its subcutaneous administration [11].

Moreover, recent studies have shown that even a factor trough level of up to 15% may not be sufficient to prevent the development of arthropathy, especially in patients with a very active lifestyle or synovitis [[12], [13], [14]]. Additionally, up to 20% to 30% of persons with severe hemophilia A and 1% to 5% of persons with severe hemophilia B develop neutralizing alloantibodies (inhibitors) directed against the exogenous factor administered in the first 20 to 50 exposures, resulting in complete or partial inactivation of replacement therapy [15,16].

However, despite the evolution of hemophilia treatment, the risk of bleeding in patients with severe disease remains high, especially in cases of trauma or major surgery, events for which it is still necessary to treat the patient with replacement or bypassing drugs (in the presence of inhibitors) [5].

Gene therapy fits into this context as a valid alternative, ensuring the long-term expression of the missing factor with levels in most cases higher than currently recommended and, in some cases, lasting for several years, leading to a significant clinical benefit and improvement in the quality of life for patients [17,18]. However, it is crucial to consider all aspects of this innovative therapy, both in terms of advantages and risks for the patient, and to plan a multidisciplinary assessment, discussion with the patient, and close monitoring of the effects and complications of the therapy, aiming for an informed and shared decision and the greatest benefit for each individual patient.

## 2 Gene Therapy in Hemophilia

Hemophilia is an ideal target for gene therapy for several reasons: it is caused by a single genetic defect, an expression of approximately 5% to 10% of the factor is sufficient to achieve a significant clinical improvement in the bleeding phenotype, and gene expression can be easily assessed by measuring correct factor levels in the plasma [17].

To deliver the missing gene into cells, the predominantly used method for monogenic disease therapy utilizes recombinant adeno-associated viruses (rAAV), characterized by a high tropism for the liver and the ability to remain predominantly in episomal circular form within hepatocytes, with low rates of integration into genomic DNA [19]. Several previous studies demonstrated that the genome of the rAAV vector does not undergo site-specific integration into the host’s DNA, nor does it alter the genetic process, but rather remains largely in episomal form in the nuclei of transduced cells [20]. However, some evidence from preclinical studies on canine models showed that in liver samples treated with canine FVIII through adeno-associated virus (AAV) 8 or AAV9 vectors, AAV could integrate into the host’s genomic DNA and in partially expanded cell clones, with 44% of integrations occurring near genes involved in cell growth. In any case, after an observation period of at least 10 years, all integrated vectors were partially removed and/or restructured, with no signs of liver dysfunction or tumor development [21,22].

Wild-type AAV is a type of nonpathogenic parvovirus characterized by a DNA replication defect [[20], [21], [22], [23], [24]]. The host’s transcriptional machinery transcribes the transgene into mRNA, which is then translated into the protein of interest [25]. AAV-based gene therapy was initially studied about 20 years ago through intramuscular injection of rAAV-FIX in persons with hemophilia B. Although the procedure was deemed safe at the time and demonstrated factor expression in muscle samples for more than 3 years, in most cases, the plasma level of FIX remained below 1% [[26], [27], [28]]. An AAV2-based gene therapy in persons with hemophilia B dating back to 2006 showed persistent FIX expression in their livers up to 2 months after administration, with no adverse events, liver toxicity, or hepatocellular carcinoma (HCC) development even after an observation period of 12 to 15 years postvector administration [29,30]. However, recently, it has been speculated that an association exists between AAV2 infection and pediatric cases of unexplained hepatitis [31].

Later, the first study with AAV8 therapy in persons with hemophilia B administered intravenously resulted in plasma FIX levels ranging from 1 to 6 IU/dL, with a reduction in bleeding episodes without the need for prophylaxis [32]. In the 6 treated patients with the highest vector dose, median FIX levels remained stable at around 5 IU/dL at 3 years and persisted even up to 8 years [33]. This strategy used a complementary DNA encoding wild-type FIX. These promising results formed the basis for further development of gene therapy by various research groups. Currently, a total of 56 gene therapy studies for hemophilia are registered on Clinicaltrials.gov (November 2024), with 12 currently in phase 3, and 3 of these products are now authorized by the Food and Drug Administration (FDA) and the European Medicines Agency (EMA) for hemophilia A and hemophilia B.

### 2.1 Hemophilia A

The first study employing liver-directed gene therapy for persons with hemophilia A based on AAV, valoctocogene roxaparvovec, administered intravenously, was reported in 2017 [34]. It is based on a rAAV5 vector, codon-optimized, using a liver-specific hybrid transcription promoter. Due to the size of the full-length FVIII gene, too large to be inserted into an AAV viral vector, a recombinant human FVIII lacking the B domain (B-domain-deleted [BDD]), which is unnecessary for the procoagulant activity of the cofactor, was chosen. The B domain was replaced with a short 14-amino acid sequence, promoting efficient intracellular cleavage [35].

In the phase 3 study (NCT03370913), a single infusion of valoctocogene roxaparvovec at 6 × 10^13^ vg/kg was administered to 134 adult males with severe hemophilia A (FVIII ≤ 1 IU/dL) without inhibitors [36]. FVIII activity, assessed with a chromogenic assay, increased on average to 41.9 IU/dL in weeks 49 to 52, leading to an 83.8% reduction in the average bleeding rate requiring treatment and a 98.6% reduction in the FVIII infusion rate. In the 2-year follow-up available for 132 patients, a reduction in the average FVIII activity level to 24.4 IU/dL was observed. At week 104, 18% of study participants had a median FVIII activity level equal to or greater than 40 IU/dL, while 24% had an activity level below 5 IU/dL [37]. Recent data from the 7-year follow-up showed a reduction of the annual bleeding rate (ABR) from baseline by 96% for the highest dose cohort at year 7 [38]. Valoctocogene roxaparvovec has already been approved by the EMA under the trade name Roctavian (BioMarin Pharmaceutical Inc©), indicated for the treatment of adult persons with severe hemophilia A without inhibitors.

A second study using a different rAAV3 vector, dirloctocogene samoparvovec (SPK-8011, NCT03003533), enrolled 18 males with hemophilia A, divided into 4 cohorts based on the vector dose administered, ranging from a minimum of 5 × 10^11^ vg/kg to a maximum of 2 × 10^12^ vg/kg [39]. In the 2-year follow-up involving 12 patients from the initial cohort, FVIII levels remained stable and ranged from 12 to 30 IU/dL, resulting in a 91.5% reduction in bleeding. However, 2 patients from the initial cohort completely lost FVIII expression due to an immune reaction to the AAV capsid unresponsive to immunosuppression with corticosteroids. The drug is currently in phase 3 development.

The study on AAV6-FVIII BDD giroctocogene fitelparvovec (NCT03061201) divided 11 patients into 4 groups based on the administered vector dosage: 9 × 10^11^, 2 × 10^12^, 1 × 10^13^, and 3 × 10^13^ vg/kg (2 patients for each of the first 3 groups and 5 patients for the fourth) [40]. Patients in the highest dose cohort reached normal FVIII levels (mean ± SD at 8 weeks, 61.5 ± 26.1 IU/dL) without bleeding events and without the need for FVIII administration at 24 weeks. After 1 year of follow-up, median FVIII levels were 50.2 IU/dL (mean ± SD, 80.1 ± 93.3 IU/dL); however, individual patient data for most patients showed a gradual decline over the 156-week follow-up period [40]. During the first year after infusion, the number of bleedings was zero, while it was 0.9 at 2 years, and 2 patients experienced a total of 3 bleeding episodes (2 traumatic; 1 unknown and 1 occurred in a target joint) requiring replacement therapy. No patient needed to resume prophylaxis. The drug is currently undergoing evaluation in a phase 3 trial.

An open-label, nonrandomized study is also ongoing with the aim of evaluating the efficacy of SPK-8016 in adult males with severe hemophilia A without FVIII inhibitors and in the absence of neutralizing anti-AAV antibodies (NCT03734588) [41]. Preliminary results showed sustained FVIII levels (6.2%-21.8%) at 52 weeks in 4 patients who received SPK-8016 at a dose of 5 × 10^11^ vg/kg. However, a total of 7 bleeding events have been reported in the first year of follow-up, 6 of which were traumatic while 1 was spontaneous. Currently, efforts are being made to optimize the immunosuppressive regimen to ensure clinically significant activity even with lower vector doses [20]. The trial is currently suspended due to an impact that is lower than expected.

In a phase 1/2 study (NCT03370172) aimed at assessing the efficacy of TAK-754, a gene therapy using a modified AAV8 was administered in 4 adult males with severe hemophilia A included in 2 incremental dosage groups [42]. The trial was later suspended due to the loss of FVIII expression in all recruited subjects following an increase in transaminase levels.

The AAVhu37 capsid vector technology (peboctocogene camaparvovec, previously known as DTX 201 or BAY2599023, Bayer’s GET8 study) was employed in a phase 1/2 study [43]. In total, 9 patients in 3 dose cohorts showed variable FVIII expression, which remained stable over up to 23 months. Patients in the 2 higher dosage groups did not require FVIII treatment from weeks 6 to 12 after therapy administration, and no spontaneous bleedings were reported as long as FVIII values remained >11 IU/dL [44]. More recently, a fourth cohort receiving a higher dose of vector (4 × 10^13^genetic copies/kg) has been added to the study design [45]. The company has not indicated any plans to discontinue the clinical program after phase 1/2. However, there is currently no indication that it will progress to phase 3.

### 2.2 Hemophilia B

Most gene therapy studies for hemophilia have been conducted on persons with hemophilia B. Currently, there are a total of 35 clinical trials on gene therapies for hemophilia B, all based on AAV therapies (clinicaltrials.gov, April 2024).

In this case, the most commonly used approach is intravenously administering an AAV vector loaded with a transgene containing a functional copy of the F9 gene with a liver-specific promoter. Several clinical studies use FIX-Padua, a variant of the F9 gene with an amino acid substitution (R338L), making it 5 to 10 times more active than the wild-type [46].

An important study evaluated the efficacy of AMT-061, or etranacogene dezaparvovec (Hemgenix, CSL Behring LLC©), which used an AAV5 vector and demonstrated maintaining high FIX activity levels for up to 24 months after injection (Trial of AMT-061 in Severe or Moderately Severe Hemophilia B Patients [HOPE-B], NCT03569891) [47]. Of the 54 participants who received the injection, 52 discontinued prophylaxis with replacement therapy; of the remaining 2, one participant with a low response level had a high titer of neutralizing anti-AAV5 antibodies, and the other received only a partial dose (10% of the planned dose) due to a hypersensitivity adverse event, then continued prophylaxis with replacement therapy [48]. At the 24-month follow-up after gene therapy, 1 (2%) participant had a 1-stage FIX activity of less than 5%, whereas 18 (33%) had FIX activity of more than 40% [49].

Another study on 3 patients treated with the same regimen showed stable FIX activity levels after 4 years of follow-up (45%), without bleeding episodes between the third and fourth years. A reduction in ABR for the cumulative follow-up period was observed, ranging from 0.22 in the third year to 0.17 in the fourth year. During 4 years of follow-up, no patients returned to continuous prophylaxis [50]. Marketed under the name Hemgenix, etranacogene dezaparvovec has been approved by both the FDA and EMA. After a single dose, FIX levels reached 30% to 40%, resulting in a true conversion of severe hemophilia B to mild and a marked reduction, nearly to zero, in bleeding events, much greater than that achieved previously in the same cases with recombinant FIX prophylaxis [47,48]. Despite the plasma FIX levels achieved and sustained over time and being higher than those obtained with gene therapy for hemophilia A, there was still significant individual variability, with some patients responding less and others responding with plasma FIX levels above the 40% cutoff, ranging from a minimum of 5% to a maximum of 99% of plasma FIX [48].

In late April 2024, the FDA approved the use of gene therapy fidanacogene elaparvovec (SPK9001; Beqvez/Durveqtix, Pfizer/Spark) for adults with hemophilia B. In the phase 3 study, the average FIX activity level after 5 years was 19.8%, with a value of 25.4% in the first year of treatment [51]. During the follow-up, no severe adverse events were observed, only some minor events, the most common being an increase in transaminases or transient myalgias. Also, 4 patients who underwent surgery during follow-up did not experience an increase in bleeding. In A Study to Evaluate the Efficacy and Safety of Factor IX Gene Therapy With PF-06838435 in Adult Males With Moderately Severe to Severe Hemophilia B (BENEGENE-2) trial, a reduction in ABR of 71% was observed up to 15 months after infusion with fidanacogene elaparvovec, with 64% of patients not experiencing a single bleeding during this period [52].

In a recent phase 1/2 study using FLT180a, an AAV3 loaded with FIX-Padua, 10 patients were recruited and treated in 4 cohorts (maximum dose, 1.5 × 10^12^ vg/kg) [53]. After therapy, high FIX levels (from 24 to 168 IU/dL at 3 weeks) were observed, which remained stable during the 1-year follow-up [54].

However, gene therapy has not always proven effective. In a study on AskBio009/BAX 335 (NCT01687608), only 1 of the 7 recruited patients achieved FIX activity exceeding 20%, which remained stable for 4 years, while in the other patients, it dropped below 20% after 5 to 11 weeks, requiring the resumption of prophylaxis [55]. The trial is currently active, not recruiting. Similarly, the effectiveness of AAVrh10 was observed only in the short term, with peaks between 12 and 20 IU/dL and subsequent loss of expression in 5 out of 6 individuals [56].

Regarding recent ongoing studies, between November and December 2022, a phase 1/2 study on a new drug, ZS801, based on AAV with a synthetic capsid, was registered in China (NCT05641610). This study is divided into a dose-escalation phase, in which 16 patients will be enrolled sequentially every 3 weeks or more between cohorts and administered with a single infusion of ZS801, and a dose-expansion phase, in which 5 more patients will be enrolled and administered ZS801. The trial is not recruiting yet.

In July 2022, the results of the phase 1 trial of BBM-H901 (NCT04135300) on 10 male patients were published, with a good response achieved during a median follow-up of 58 weeks in the absence of severe adverse events. The most common adverse events were fever and increased transaminases. In 2 participants, an increase in alanine aminotransferase (ALT) and aspartate aminotransferase was observed concurrently with a decrease in FIX [57]. The trial is still ongoing as a phase 1 study.

### 2.3 Duration of expression

Concerning hemophilia A, most trials on various available therapies have shown a persistence of factor levels exceeding 10 IU/dL up to 2 years after treatment in most patients, although with a progressive decrease in these levels over time. Rare isolated cases of complete loss of expression have been associated with unpredictable immune reactions to the AAV capsid that are not responsive to immunosuppression with corticosteroids [39,42,58,59]. A recent study on patients treated with valoctocogene roxparvovec followed for 7 years after administration, demonstrated a loss of expression in 2 out of 13 patients in the seventh year of follow-up [38].

Regarding hemophilia B, efficacy has been demonstrated to be even higher than observed for hemophilia A, although still with marked individual variability. Recent studies have also shown, in this case, a persistence of expression of the missing factor that remains stable up to 3 years after therapy [26,47,60,61].

### 2.4 Limits and safety profile

While gene therapy has demonstrated a significant reduction in bleeding episodes, and these positive results have led to approval by the FDA and EMA, there are some limitations and many safety aspects to consider. One disadvantage of therapy is the unpredictability of the levels of the deficient coagulation factor achieved. On the other hand, the only reported thrombotic event with gene therapy is a thrombotic occlusion of the arteriovenous fistula requiring anticoagulant therapy in one of the patients treated with FLT180a, who, however, had a high expression of FIX (>200 IU/dL) [54].

In addition, the presence of preexisting neutralizing antibodies against AAV, widespread in the general population, may prevent cell transduction, with consequences for the efficacy of gene transfer [[62], [63], [64]]. The formation of neutralizing antibodies also occurs after the initial administration of gene therapy, interfering with the initiation of expression by inhibiting transduction and preventing subsequent readministration of gene therapy with the same vector. The immune response to the vector may necessitate additional strategies, such as switching to alternative vectors or using immunomodulatory drugs, to overcome this limitation and enhance the effectiveness of gene therapy.

The presence of neutralizing antibodies may prevent subsequent administration in the case of a reduction or loss of deficient factor expression, although strategies to reduce antibody titers or eliminate them before a new treatment are being explored [65]. For these reasons, preexisting neutralizing antibodies remain an exclusion criterion in clinical studies. However, in the AMT-061 study, individuals with preexisting neutralizing antibodies against AAV5 were also included, only one of whom showed no therapeutic response, showing that even relatively high antibody titers may allow FIX expression [66]. The role of nonneutralizing antibodies, on the other hand, is less clear [67]. Currently, various nonstandardized tests are used to detect total anti-AAV antibodies and transduction inhibitors, which, most of the time, are neutralizing anti-AAV antibodies. The lack of standardization in anti-AAV antibody testing poses a significant challenge by complicating meaningful comparisons across gene therapies. To address this, international scientific organizations, including the International Society on Thrombosis and Haemostasis Scientific and Standardization Committee Working Group on Gene Therapy, are actively developing standardized protocols. Their efforts include surveying current laboratory techniques, conducting interlaboratory comparisons with calibrated samples, and supporting the incorporation of International Organization for Standardization (ISO) standards. This initiative aims to harmonize testing methods, enhancing comparability and reliability in patient evaluations and ultimately advancing clinical practices and outcomes in AAV-based gene therapies [68].

Regarding other safety considerations, more than two-thirds of treated cases showed an increase in serum transaminases in follow-up, requiring corticosteroid administration. With valoctocogene roxaparvovec, among the reported adverse events, the most common were increased transaminases, headache, and nausea. Every study participant experienced at least 1 adverse event; of these, 8.2% had a severe increase in ALT, and 5 patients reported a treatment-related serious adverse event, particularly syncope, rash, hypersensitivity, and anaphylactoid reaction. All severe adverse events resolved, as did 96.2% of ALT increases [36]. On the other hand, no participant withdrew from the study due to adverse events or developed FVIII inhibitors, and no treated patient died from causes related to the gene therapy. With giroctocogene fitelparvovec, the most common treatment-related adverse events were increased transaminase levels, episodes of fever, and tachycardia. No patient developed FVIII inhibitors [40]. Even with dirloctocogene samoparvovec (SPK-8011), a modest increase in ALT was observed in 7 patients [39]. With etranacogene, an increase in transaminases was observed in approximately 20% of cases, a lower percentage than that seen with gene therapies for hemophilia A. Additionally, the duration of transaminase elevation was generally shorter in these cases [48]. BBM-H901 proved safe in the 12 enrolled patients following prophylactic corticosteroid use 1 year after administration [57]. In the phase 3 Study to Evaluate the Efficacy and Safety of PF-07055480 / Giroctocogene Fitelparvovec Gene Therapy in Moderately Severe to Severe Hemophilia A Adults (AFFINE) study on giroctocogene fitelparvovec, deep vein thrombosis was observed in a patient with high FVIII levels after dosing. The study was placed on temporary hold to implement a protocol amendment to provide guidelines for the clinical management of patients with FVIII levels >150%. These observations highlight the importance of monitoring and managing FVIII levels not only to monitor response to treatment but also to avoid excess FVIII.

## 3 Hepatological Complications of Hemophilia Gene Therapy

The most common side effect of gene therapy is the increase of transaminases, particularly ALT, which inconstantly correlates with the loss of factor expression. ALT increase has been observed more frequently in gene therapy studies for hemophilia A (82%) compared with hemophilia B (40.4%) [36,69,70]. The mechanisms of hepatocyte damage have not yet been clearly defined. Initially, some evidence was in favor of a direct cell-mediated response against the antigens of the viral vector capsid expressed by hepatocytes or an increase in cellular stress at the endoplasmic reticulum level [71,72]. However, a clear cause-effect relationship between hepatocellular damage and loss of factor expression has not been demonstrated. Notwithstanding, transient immunosuppressive therapy was frequently administered in clinical trials for any increase of transaminases, and corticosteroids were the most common regimen of choice. Alongside this reactive approach, therapeutic prophylaxis with glucocorticoids was also used (eg, in A Phase I/II, Open Label, Multicentre, Ascending Single Dose, Safety Study of a Novel Adeno- Associated Viral Vector (FLT180a) in Patients With Haemophilia B [B-AMAZE] study), sometimes in combination with tacrolimus [54].

In this context, it is worth noting that any immunosuppression scheme was primarily indicated to preserve transgene expression since the level of transaminase elevation observed in the trials was often mild and never associated with liver failure. Moreover, the few available liver biopsy data in patients treated with valoctocogene roxaparvovec 2.6 to 4.1 years after gene therapy failed to demonstrate any histological alteration associated with immune-mediated damage deserving steroid therapy [71]. In preclinical models, the mechanism regulating the variability of FVIII expression remains unclear, although it does not seem to be modulated by steroid therapy [73,74]. Finally, although the increase of transaminases seemed restricted to the phase close to gene transfer, ALT increases of unclear significance have been reported up to 3 years after treatment. Interestingly, none of the late ALT increases in the third year needed any treatment [75]. Despite the lack of compelling evidence mentioned above, immunosuppressive therapy with corticosteroids has been actively implemented in clinical trials. In the phase 3 study on valoctocogene roxaparvovec, the median duration of immunosuppression was 230 days, significantly contributing to the onset of corticosteroid therapy-related side effects [36]. The need for immunosuppression was relatively lower in gene therapy studies for hemophilia B, with a median duration of 78 days [26,69]. Today, based on the experience of the trials, the most recommended schedule of treatment would be a reactive strategy, not a preventive one, starting with oral prednisone 60 mg per day or an equivalent dose of corticosteroids for the first 2 weeks by tapering in the third week in case of good biochemical response. For those not achieving this goal, 1.2 mg/kg per day of prednisone could be considered. In our opinion, the low number of patients treated with high doses and/or different routes of administration of corticosteroids, the low number of patients addressed to alternative immunosuppressants (eg, budesonide, tacrolimus, and mycophenolate), and the lack of a comparison with the placebo, the knowledge gap on the pathogenic mechanism causing ALT increase are all valid reasons to limit the adoption of immunosuppressants to preserve transgene expression based on a case-by-case decision. Generally, the clinical trials shared a highly conservative ALT threshold as a trigger for therapy. In particular, for hemophilia A, the threshold was 1.5 times the baseline value. When translating this approach to real-world clinical practice, relying on a single laboratory for transaminase measurements is not recommended due to potential fluctuations between normal and abnormal values [76]. Therefore, using the average of at least 2 pretherapy values as a baseline is advisable [77,78]. Any transaminase elevation above baseline should be reviewed by a multidisciplinary team, including hepatologists and hematologists. In our opinion, the 1.5 increase in baseline with ALT remaining in the normal range is questionable and would need further exploration. The decision for immunosuppressive therapy should be taken case-by-case after an exhaustive work-up to exclude any alternative cause of transaminase increase (eg, viruses, hepatotoxic medications/substances, inclusive herbal products, and alcohol) and finally shared with patients to best balance the benefit/risk ratio of this therapy. In some cases, the hepatologist, particularly for persistent elevation of ALT, could propose a liver biopsy, and the transjugular route should be preferred to minimize the procedure-related risk of hemorrhage [77,79]. Further exploration of the mechanism of loss of FVIII and, potentially, no intervention arm while evaluating potential immunosuppressive effects are hugely awaited to shed light on this bench-to-bedside dilemma. The other feared liver-related complication of gene therapy is genotoxicity, specifically the development of HCC as a result of potential integration events at the oncogenes level. To date, only 1 case of HCC has been described during the 1-year follow-up after gene therapy for hemophilia B [80]. The patient was more than 65 years old and had a history of hepatitis C virus (HCV) eradication 3 years before gene therapy with direct-acting antivirals, previous contact with hepatitis B virus (HBV), and steatosis on biopsy analysis. At enrollment, the patient had been evaluated through ultrasound with no evidence of liver lesions, and blood tests (ALT, aspartate aminotransferase, and alpha-fetoprotein) were consistently within the normal range. Subsequent analyses included more than 220,000 cells from the biopsy sample of the neoplastic lesion, identifying 60 cells with random integration phenomena, which were not associated with HCC development. Moreover, the complete genetic sequencing found anomalies in chromosomes 1 and 8 and mutations in TP53 and other potential oncogenes [70]. All these observations led to consider an unlikely causal relationship with gene therapy. The concern about genotoxicity resides in the risk of AAV genome integration, which is not negligible despite remaining primarily in episomal form [81,82]. A recent study demonstrated the presence of fragments of wild-type AAV2 genome integrated near known proto-oncogenes in a small percentage of HCC samples [83]. Furthermore, biopsy data obtained 2 to 4 years after gene therapy administration showed the presence of episomal and nonintegrated vector DNA in the genome [73]. Available studies on murine animal models and larger animal models suggest a relatively low risk of tumorigenesis with AAV-based vectors, although 2 studies on animals receiving gene therapy via AAV for diseases other than hemophilia reported an increase in HCC [84]. In any case, in the gene therapy trial for hemophilia with the most extended 15-year follow-up, no long-term liver toxicity, including HCCs, was observed [30]. Considering all these aspects, a thorough evaluation of liver health and inclusion criteria during screening is deemed essential, with continuous monitoring during follow-up and inclusion in national and international registries of patients undergoing gene therapy (Table 1) [70,85].

**Table 1:** Hepatological exclusion criteria adopted in the summary of product characteristics based on clinical trials.

Hepatological exclusion criteria	Hemophilia A *Valoctocogene roxaparvovec*	Hemophilia B *Etranacogene dezaparvovec*
Laboratory	ALT/AST/GGT/total bilirubin > 1.25× or INR ≥ 1.4 on at least 2 measurements within 3 mo	ALT/AST, 2 assessments within 3 mo ALP/total bilirubin once within 3 mo Any alterations to be evaluated by a hepatologist
Virological	Uncontrolled chronic hepatic infection	Uncontrolled HBV or HCV infection
Cirrhosis/advanced fibrosis evaluation	Ultrasound/elastography within 3 mo OR laboratory tests of fibrosis	Ultrasound and liver stiffness by Fibroscan (cutoff 9 kPA) within 6 mo
Hepatological evaluation	Recommended	Considered if there is any alteration
Alcohol	To be stopped for 1 y, then minimal	Not properly specified (case-by-case evaluation)

ALP, alkaline phosphatase; ALT, alanine aminotransferase; AST, aspartate aminotransferase; GGT, gammaglutamiltransferase; HBV, hepatits B virus; HCV, hepatitis C virus; INR, international normalized ratio.

Clinical gene therapy trials in hemophilia had very heterogeneous hepatological exclusion criteria without a proper “liver-health” comprehensive assessment. As a consequence, hepatological assessment is also not homogenously defined for the 2 gene therapies approved in the European Union. Furthermore, we recently showed that a nonnegligible proportion of HCV-cured persons with hemophilia remain at risk for advanced fibrosis/cirrhosis or present risk factors for liver disease progression [86]. There remain some open questions in the hepatological management of persons with hemophilia eligible for gene therapy, such as the role of steatosis or low-intermediate fibrosis in the risk of hepatotoxicity, the optimal management of patients with hepatitis B virus surface antigen (HBsAg) positivity on nucleoside analog therapy, and the risk of hepatitis B reactivation in patients with ongoing immunosuppressive therapy and occult HBV infection. In Table 2, we propose some practical recommendations for liver safety prior to gene therapy administration based on our experience and discussion with experts worldwide [77,79]. We believe that active cooperation between hepatologists and hematologists is mandatory to answer all the new questions posed by this innovative liver-directed treatment for hemophilia [70,87].

**Table 2:** Practical recommendations for liver safety before gene therapy: from clinical trials to real life.

Type of evaluation	Time before GT	Assessments	Aims
Laboratory	3 mo	AST/ALT (mean of at least 2 measurements) GGT/ALP, bilirubin, albumin, alpha-fetoprotein, and full blood count	Set AST/ALT baseline as a reference point for any increase postinfusion Exclude active hepatocellular/cholestatic liver damage Detect signs suggestive of advanced liver disease Calculate APRI^a^ and FIB-4^b^ as noninvasive tests of fibrosis
Virological assessment	3 mo	Screening HBV/HCV/HIV: HBsAg, HBsAb, HBcAb, HCV-Ab, and HIV-Ab If HBsAg+: HDV-Ab and HBV DNA If HCV-Ab+: HCV RNA	Exclude active HCV/HBV (±HDV) infections
Imaging and liver stiffness measurement	6 mo	Liver ultrasound (experienced operator) Liver stiffness by Fibroscan Level 2 imaging (CT/MRI) if required after hepatological evaluation	Evaluate signs of advanced fibrosis/cirrhosis and malignancy
Hepatological visit	3 mo	Check for any potential risk factor of chronic liver damage by including alcohol and metabolic features and liver biochemistry Patient education	Exclude active chronic liver damage (whatever the etiology) Exclude advanced fibrosis/cirrhosis and malignancy Explain: 1. Potential hepatological short- and long-term adverse effects of GT 2. Need for periodical monitoring 3. Counseling for any substance/medication at risk of inducing liver damage after assumption

ALP, alkaline phosphatase; ALT, alanine aminotransferase; APRI, AST to platelet ratio index; AST, aspartate aminotransferase; CT, computed tomography; FIB-4, fibrosis-4; GT, gene therapy; GGT, gammaglutamiltransferase; HBV, hepatits B virus; HBcAb, hepatitis B virus core antibodies; HBsAb, antibodies against hepatitis B virus surface antigen; HBsAg, hepatitis B surface antigen; HCV, hepatitis C virus; HCV-Ab, antibodies against hepatitis C virus; HDV, hepatitis D virus; HDV-Ab, hepatitis D virus antibodies; HIV-Ab, human immunodeficiency virus antibodies; INR, international normalized ratio; MRI, magnetic resonance imaging.

a APRI formula: (AST U/L)/(AST upper limit of normal U/L)/(Platelets 10^9^/L).

b FIB-4 formula: (Age × AST U/L)/(Platelets 10^9^/L × √[ALT U/L]).

## 4 Need for an Expert Multidisciplinary Gene Therapy Team

The shift in the management paradigm of persons with hemophilia implies new challenges for the centers administering gene therapy and those involved in follow-up and adverse event management. In this regard, the European Association for Haemophilia and Allied Disorders (EAHAD), in collaboration with the European Haemophilia Consortium, has recently established a working group for the accreditation of facilities intending to administer gene therapy (hub center) and for those monitoring the success and long-term safety of therapy (spoke center). The aim is to ensure quality and improve the management of bleeding disorders in Europe, supporting European hemophilia centers with a new accreditation model [88]. European guidelines for the certification of hemophilia centers have been updated to accreditation guidelines, encompassing all the necessary features in terms of facilities, laboratories, and expert personnel for the optimal management of new therapeutic options, including the hub-and-spoke model for gene therapy administration. According to EAHAD, hub centers must be equipped and certified to order, store, prepare, and administer gene therapy drugs; have experience gained in previous gene therapy clinical studies or experts who can provide gene therapy expertise or available mentorship programs; have diagnostic testing availability for the gene therapy administration program and its follow-up; collaborate with other certified hemophilia centers in the EAHAD hub-and-spoke network; have experience in recognizing and managing gene therapy complications; establish close collaboration with hepatologists and immunologists; and have drafted protocols for different immunosuppression strategies [88].

In the context of this new model, it is crucial to consider the evolution of the multidisciplinary team in managing persons with hemophilia [18,89,90]. This team should include a hematologist, hepatologist, pharmacist, physiotherapist, nurse, and psychologist, all with adequate training in gene therapy. The hematologist’s role will involve informing and assessing the patient’s eligibility in clinical and psychological terms, assisted by the psychologist, who will support the patient even after drug administration. The role of the hepatologist in the multidisciplinary team of the hemophilia centers is crucial in all phases of gene therapy administration, from the selection phase to the treatment moment to the long-term monitoring phase. The experienced nurse will coordinate all these activities, informing the patient, caregivers, and family members, ensuring patient adherence to the monitoring program, including lifestyle adjustments, and assisting the hematologist in observing and managing any adverse events to immunosuppressive therapy if necessary [90].

It will be crucial to address early discussions with patients regarding some critical aspects related to gene therapy [91]. For example, the risk of transmitting the transgene to the germline. For this reason, treated patients are required to use effective contraceptive methods for at least 1 year after therapy, even though data from animal models appear reassuring [92]. The need to modify lifestyle by abolishing and then reducing alcohol consumption should also be discussed, as well as the need to start immunosuppressive therapy in case of transaminase elevation after therapy administration, preparing the patient for the psychosocial impact of a change in disease management [90].

In the multidisciplinary care of persons with hemophilia undergoing gene therapy, the physiotherapist’s role is essential both before and after treatment. Before therapy, physiotherapists conduct a comprehensive musculoskeletal assessment and create individualized plans to enhance muscle strength, joint stability, and mobility while minimizing bleeding risk. Patient education on joint protection is also prioritized to maintain physical health and reduce the risk of joint damage. After gene therapy, with adequate bleeding prophylaxis, physiotherapists can focus on progressive strengthening and targeted rehabilitation to restore range of motion, enhance mobility, and correct gait issues to support long-term joint health and quality of life.

The presence of a figure like an internist or rheumatologist, an expert in clinically and ultrasound-monitored joint health before and after therapy administration, completes the multidisciplinary team. Over the years, it has been realized that outcomes based on patient-reported outcomes, such as the ABR, are not always accurate, as patients often do not remember the number of bleeding events correctly unless they keep a diary. The introduction of point-of-care joint ultrasound has allowed for a differential diagnosis of acute joint pain, recognizing intra-articular bleeding early, which has irreversible consequences on cartilage and synovial membrane [3,93], and detecting the presence of synovitis, a sign of inadequate prophylaxis, or advanced osteochondral damage that could cause acute or chronic joint pain.

Joint ultrasound also allows monitoring the progression of hemophilic arthropathy over time and comparing the severity of synovitis and osteochondral damage before and after changing the prophylaxis regimen and, therefore, even in the case of gene therapy administration, applying specifically designed scores such as the Hemophilia Early Arthropathy Detection with Ultrasound (HEAD-US) score [94].

In the future, valuable assistance in the continuous monitoring of these patients, even remotely, will be provided by artificial intelligence algorithms [95] and telemedicine systems utilizing portable ultrasound probes [96,97].

## 5 Ethical Considerations

The comprehensive evaluation of risks and benefits has implications for both informed consent in clinical studies and treatment [98]. There has been repeated discussion about the actual level of understanding among potential participants regarding the nature and purposes of gene therapy studies [[99], [100], [101], [102], [103]]. More recently, the importance of informed consent has been emphasized even in a treatment setting, emphasizing that it should be a process rather than a single event [104].

Specifically, issues such as the unpredictability of factor expression over time and duration, considering the risk of losing expression due to a cell-mediated immune response requiring immunosuppressive therapy, need to be addressed. Some patients may perceive a loss of their identity, feeling completely "cured" and losing their sense of belonging to a group of patients.

In order to provide accurate and comprehensive information regarding safety, efficacy, costs, procedure, and postadministration monitoring, a shared decision-making tool has been proposed to allow comparison with other available therapeutic options [67,105].

It is important to note that children under 18 years of age have not been part of current phase 3 clinical trials. There is a lower age limit for obtaining efficacy and duration, given the higher turnover of hepatocytes during childhood, with a progressive loss of episomal gene expression over time. It is possible that this therapy is effective in adolescents, and in the future, the minimum age to access this treatment may be expanded [106]. In addition, the role of parents in giving consent to an irreversible treatment in an underage is still a matter of debate.

The issue of costs of gene therapy must also be discussed and carefully considered, given the observed increase in expenditure in the Italian public health system in recent years due to the progress already discussed. There are no examples of how their cost is managed in countries with a universal healthcare system like ours and the UK. Therefore, it is important in the future to establish a cost-effectiveness target to assess whether there is a positive balance.

Certainly, the number of patients actually treated will be limited, both because a significant proportion is excluded from currently limited indications (children, individuals with inhibitors, and carriers of anti-AAV antibodies, especially for hemophilia A) and because this potentially curative approach will not be required by all eligible patients.

## 6 Conclusions

In conclusion, gene therapy adds to the many therapeutic alternatives for persons with hemophilia. The process of patient information and assessment of suitability for treatment with this therapy will require a shared effort within the multidisciplinary team among different specialists and within the hub-and-spoke network of hemophilia centers to ensure the safety and efficacy of this therapy with high potential in terms of benefit for the patient and cost-effectiveness ratio for the healthcare system.
